# Ag Nanoparticles Sensitized In_2_O_3_ Nanograin for the Ultrasensitive HCHO Detection at Room Temperature

**DOI:** 10.1186/s11671-019-3213-6

**Published:** 2019-12-05

**Authors:** Shiqiang Zhou, Mingpeng Chen, Qingjie Lu, Yumin Zhang, Jin Zhang, Bo Li, Haitang Wei, Jicu Hu, Huapeng Wang, Qingju Liu

**Affiliations:** 1 School of Materials Science and Engineering, Yunnan Key Laboratory for Micro/nano Materials & Technology, Yunnan University, Kunming, 650091 China; 2 Institute of Applied Physics and Materials Engineering, University of Macau, Macau SAR, China

**Keywords:** In_2_O_3_, Ag loading, HCHO detection, Gas sensors

## Abstract

Formaldehyde (HCHO) is the main source of indoor air pollutant. HCHO sensors are therefore of paramount importance for timely detection in daily life. However, existing sensors do not meet the stringent performance targets, while deactivation due to sensing detection at room temperature, for example, at extremely low concentration of formaldehyde (especially lower than 0.08 ppm), is a widely unsolved problem. Herein, we present the Ag nanoparticles (Ag NPs) sensitized dispersed In_2_O_3_ nanograin via a low-fabrication-cost hydrothermal strategy, where the Ag NPs reduces the apparent activation energy for HCHO transporting into and out of the In_2_O_3_ nanoparticles, while low concentrations detection at low working temperature is realized. The pristine In_2_O_3_ exhibits a sluggish response (R_a_/R_g_ = 4.14 to 10 ppm) with incomplete recovery to HCHO gas. After Ag functionalization, the 5%Ag-In_2_O_3_ sensor shows a dramatically enhanced response (135) with a short response time (102 s) and recovery time (157 s) to 1 ppm HCHO gas at 30 °C, which benefits from the Ag NPs that electronically and chemically sensitize the crystal In_2_O_3_ nanograin, greatly enhancing the selectivity and sensitivity.

## Introduction

All kinds of hazardous volatile organic compounds (VOCs) gases in indoor and outdoor, such as HCHO, ethanol, acetone, benzene, methanol, and toluene, are routinely and daily emitted from agriculture and industrial processes, or released as vehicle exhaust emissions [1]. VOCs, such as HCHO, are harmful to human health and the environment when their concentrations are above a critical threshold, sometimes as low as parts-per-million (ppm) levels [2, 3]. For safety reasons, anything out of limits in HCHO storage systems, appliances, and vehicles, as well as the entire internal environment infrastructure, must be detected immediately [4–6]. The ever-increasing attention concerning indoor and outdoor air quality and workplace safety has brought out the steady development of the gas sensors market over the past few years, and therefore, gas sensors are expected to attain wider application [7–9]. Therefore, formaldehyde sensors will play a critical role on account of formaldehyde’s extensive carcinogenicity range in air [10, 11].

Metal oxide semiconductor based on chemiresistors, mainly including In_2_O_3_ [12–14], WO_3_ [15–17], SnO_2_ [18, 19], ZnO [20, 21] and LaFeO_3_ [22–24], is an outstanding technique for detecting VOCs, due to its unique advantages in terms of low cost, good sensitivity, fast response/recovery time, and a large number of gases detected [25]. However, traditional gas sensors based on metal oxide semiconductors usually have a high working temperature of 150–400 °C, which may decrease the sensor stability and life. In addition, high operating temperature leads to high power consumption, which is an important parameter for the new generation of battery-loaded wireless sensors [26, 27]. However, this can be reversed when the sensing materials are elaborately designed. A typical method used for lowering working temperature is the surface modification of the semiconductor metal oxide with noble metals such as Ag [28, 29], Pt [30], and Pd [31, 32] or different metal oxides [26]. By either chemical sensitization or electronic sensitization, one can modify the semiconductor surface with various metal promoters to achieve an effective room-temperature sensor material. Outstanding sensing performance is attributed not only to the sensitizer effect of noble metals but also to the synergistic effect of large surface area, appropriate particle size, and abundant mesoporous surface of nanostructure [15, 20, 23, 33].

In_2_O_3_ is an important n-type semiconductor with about 3.6 eV wide band gap and has been widely studied owing to its high catalytic activity and electronic properties [34, 35]. Unfortunately, the pure In_2_O_3_ as sensing material possessing simply poor selectivity and a high response can hardly be obtained at low temperatures, which restricts its further application. To further enhance its sensing properties, In_2_O_3_ has been modified by noble metals [36], metal ions [37], and carbon materials [38]. Composites of multi-phase semiconducting metal oxide nanostructures have also been frequently reported [39]. To date, few researches have been carried out on the gas-sensing properties of In_2_O_3_ sensor to HCHO. Wang et al. [29] reported that the Ag-loaded In_2_O_3_ hierarchical nanostructure sensors showed fast response (0.9 s), recovery (14 s), and high response (11.3) towards 20 ppm HCHO at 240 °C. Dong et al. [40] reported that the as-synthesized 3 wt%Ag-functionalized In_2_O_3_/ZnO samples exhibited high response of about 842.9 towards 2000 ppm HCHO at operating temperature of 300 °C. Currently, formaldehyde gas sensors have been reported to require higher operating temperatures. Zhang et al. [28] have reported the results of formaldehyde gas-sensing tests, which revealed that a sensor based on 6%-Ag/Ni_5.0_In exhibits ultra-high sensitivity (123.97) toward 100 ppm of formaldehyde at a lower operating temperature (160 °C). Wang et al. [33] reported that the graphene oxide in situ modified two-dimensional SnO_2_ nanosheets with in-plane mesopores was utilized as the sensing material and that the sensor response was larger than 2000 toward 100 ppm HCHO at 60 °C. The problem that formaldehyde gas sensors with high sensitivity and high selectivity to low concentration HCHO at room temperature have remains unsolved.

In this work, we report a high-response formaldehyde gas sensor that operates at room temperature, which is prepared with In_2_O_3_ nanograin sensitized by Ag nanoparticles. The comparative study of HCHO gas detection between pure and Ag-loaded In_2_O_3_ nanoparticles was investigated, and the influence of Ag loading on sensing performance was revealed. The results show that 5%Ag-In_2_O_3_ sensor exhibits an excellent response of 1670 to 5 ppm HCHO at 30 °C and a ultra-low detection concentration of 0.05 ppm (to which the response value is 3.85). Simultaneously, the 5%Ag-In_2_O_3_ sensor also presents superior selectivity and stability, all of which reaches the level of metal oxide sensors.

## Methods

### Sample Preparation

The pure In_2_O_3_ was synthesized through dissolving 6 mmol In(NO_3_)_3_.4.5H_2_O (99.99%, Aladdin), and 24 mmol urea (99%, Aladdin) in 45 mL of deionized water; the mixture was kept in a 50-mL polyethylene reaction pot at 140 °C for 16 h and then cooled to room temperature. The prepared sediment was washed with ethyl alcohol for three times, dried for 20 h at 70 °C, and calcined for 2 h at 600 °C in pure nitrogen flow with a heat rate of 5 °C min^−1^. The pure In_2_O_3_ was dissolved in deionized water being stirred for 20 min, and then AgNO_3_(99.8%, Sigma-Aldrich) was added to transparent solution. Under magnetic stirring, the freshly prepared NaBH_4_(98%, Aladdin) solution was drop by drop into the above mixture solution. After being stirred, the as-made sediments of Ag-loaded In_2_O_3_ were collected through centrifugation, washed with absolute ethanol for three times, and dried in air at 60 °C for 12 h. Finally, yellowish nanotructural In_2_O_3_ samples were obtained. To study the effect of the Ag loading ratio on the gas sensing response, various contrast composites with different Ag loading rates (1 wt%, 3 wt%, 5 wt%, and 7 wt%) were prepared and named 1%Ag-In_2_O_3_, 3%Ag-In_2_O_3_, 5%Ag-In_2_O_3_, and 7%Ag-In_2_O_3_, respectively.

### Characterization

The X-ray powder diffraction (XRD) of the prepared products was conducted on a D/max-2300 diffractometer (Rigaku Corporation; 35 kV) in a scanning range of 10–90° at a rate of 2°min^−1^ with Cu Kα1 radiation (l = 1.540 Å). X-ray photoelectron spectroscopy (XPS) was carried out on a K-Alpha+ spectrometer with Al Kα excitation (Thermo Fisher Scientific Co. Ltd; 1486.6 eV) to observe the chemical binding states of each element. The morphology of the samples was recorded by scanning electron microscopy (SEM, Thermo Fisher Scientific Co. Ltd.). The elemental composition was performed by SEM equipped with an energy dispersive X-ray spectroscopy (EDS) detector. Transition electron microscopy (TEM) and high-resolution transmission electron microscopy (HRTEM) of the size and crystallinity of the grain were performed by a JEM-2100 microscope (JEOL Co. Ltd.) operating at 200 kV. The N_2_ adsorption–desorption analysis of the obtained samples was collected on Beth equipment (Bestech Instrument Technology Co. Ltd.) at liquid nitrogen temperature.

### Sensor Fabrication and Sensing Test

**Fig. 1:**
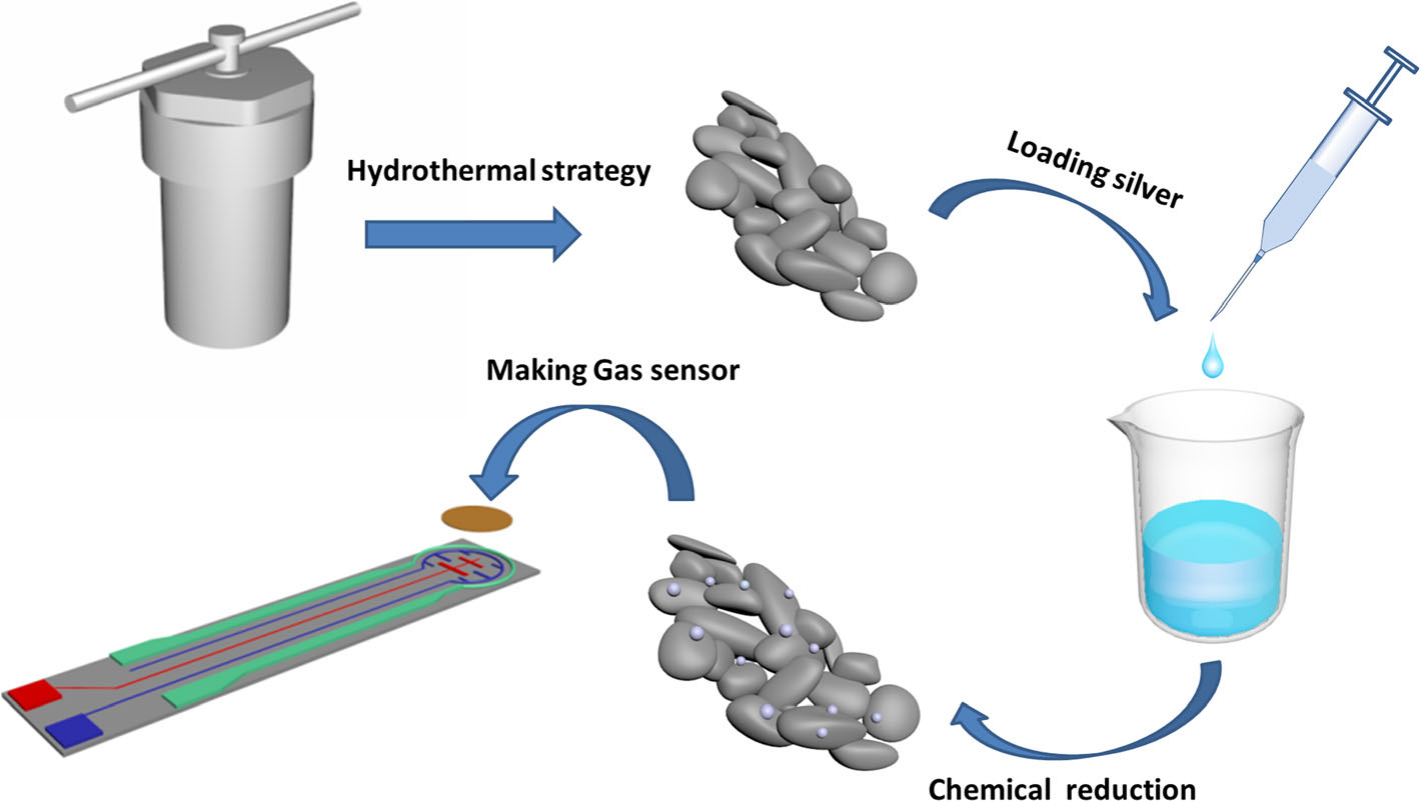
Schematic illustration of the preparation of Ag-functionalized In_2_O_3_ nanoparticles and screen printing

In the as-prepared of gas sensing materials (pure In_2_O_3_, 1%, 3%, 5%, and 7% Ag-loaded In_2_O_3_), 2 mg gas-sensing material samples were mixed with 2 mg printing oil in mortar, which was ground for 1 min in agate mortar for forming a uniform mash. The mash sensing materials were screen-printed with a mesh on the outer surface of substrate and dried at 60 °C for 10 min in a drying oven. The gas sensing material forming on the surface of substrate has a thickness of about 10 mm. Figure 1 presents the schematic diagram of the gas sensor. Finally, the devices were sintered at 400 °C for 2 h in an electric furnace to ensure its stability. Afterwards, the sensing properties were evaluated by HCRK-SD101 gas sensing analyzer (Wuhan HCRK Technology Co. Ltd.) at the relative humidity of 16 ± 10%. The prepared sensors were installed in the test chamber (2.7 L) and then injected with different concentrations of tested gas by a micro syringe. The response of the gas sensor can be defined as the ratio of the resistance value Ra to the resistance value Rg, where Ra and Rg refer to the resistance in air and target gas, respectively [41]. Response and recovery times refer to the time required to achieve 90% of the maximum sensing value during adsorption and desorption.

## Results and Discussion

### Morphology and Structure Characterization

**Fig. 2:**
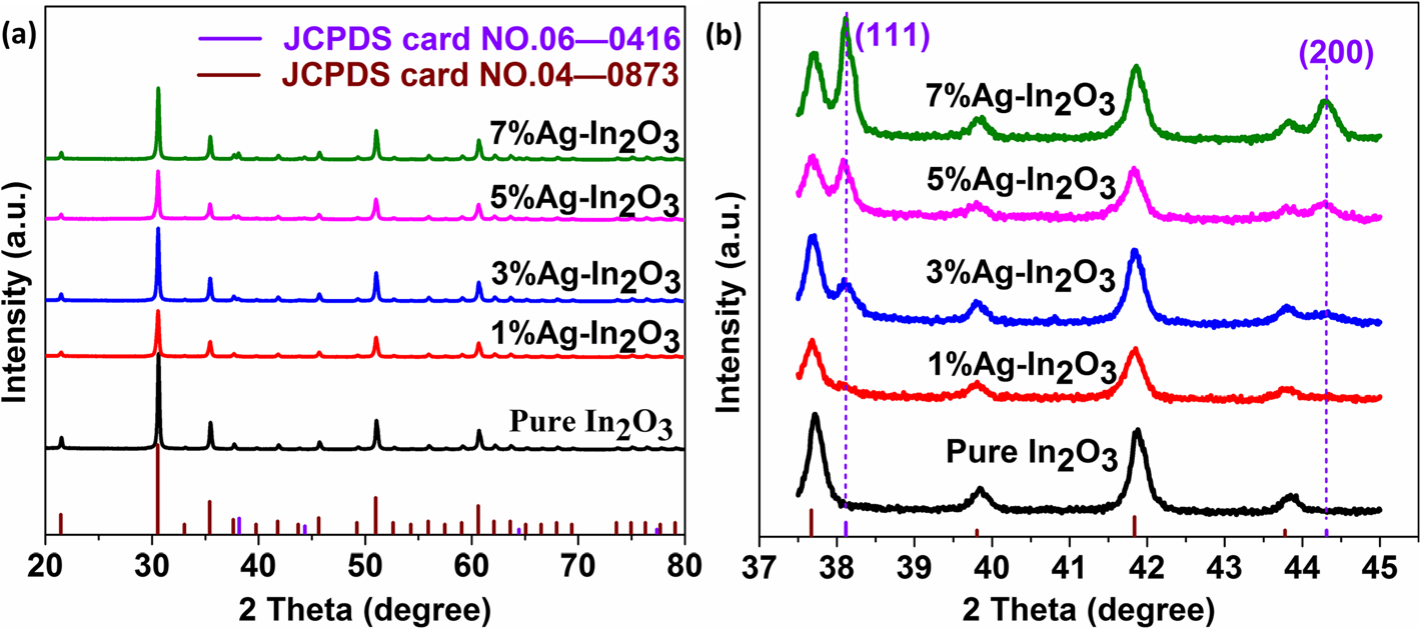
**a** XRD patterns of pure In_2_O_3_, 1%Ag-In_2_O_3_, 3%Ag-In_2_O_3_, 5%Ag-In_2_O_3_, and 7%Ag-In_2_O_3_ samples. **b** Corresponding high magnification of the 111 and 200 peaks of the samples

The crystal phase of the pure and Ag-loaded dispersed In_2_O_3_ were investigated utilizing XRD. XRD patterns of the pure and Ag-loaded dispersed In_2_O_3_ were shown in Fig. 2. It can be seen from Fig. 2a that the diffraction peaks of the In_2_O_3_ sample are similar according to the JCPDS card NO. 06-0416, which can be assigned to the cubic structure of In_2_O_3_. The diffraction peaks of In_2_O_3_ samples are located at 2y of 30.58, 35.46, 51.03, and 60.67, which are ascribed to the 222, 400, 440, and 622 planes, respectively. For Ag-loaded In_2_O_3_ samples, in Fig. 2b, the XRD curves corresponding to 1%Ag-In_2_O_3_, 3%Ag-In_2_O_3_, 5%Ag-In_2_O_3_, and 7%Ag-In_2_O_3_ are similar to those pure In_2_O_3_, indicating that the crystalline phase of In_2_O_3_ is barely influenced during the surface functionalization process. As the loading amount of Ag is increasing, the diffraction peaks of 200 and 111 agreeing with Ag (JCPDS card NO.04-0873) can be gradually detected by small bulges and continuously shift to larger angles. No impurity phase was examined from the XRD patterns, which further confirmed the prominent purity of the samples.

**Fig. 3:**
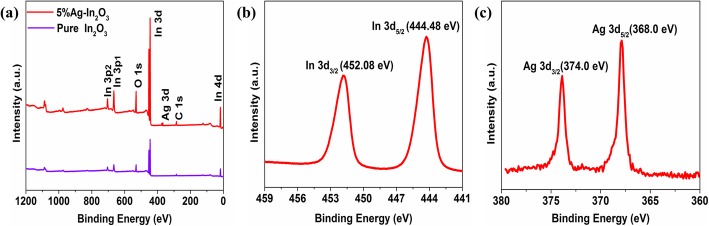
**a** XPS spectrum of pure In_2_O_3_ and 5%Ag-In_2_O_3_ samples. **b** In 3d spectrum. **c** Ag 3d spectrum

To further demonstrate the component and the chemical states of the as-synthesized samples in the surface region, XPS was presented. The full XPS spectra (Fig. 3a) reveal that the 5%Ag-In_2_O_3_ sample mainly contains In, O, Ag, and C elements. The presence of elemental C in the spectrum is due to the binding energy of C 1 s, which is usually used as an internal reference in the spectrum during XPS measurements. All XPS spectra were calibrated with a C1s peak of 284.8 eV, as shown in Fig. 3. The high-resolution In 3d XPS spectrum can be fitted with two strong peaks with binding energies at 452.08 eV (In 3d_3/2_) and 444.48 eV (In 3d_5/2_) in Fig. 3b. Compared with the reported In 3d_5/2_ (443.60 eV) signal of metallic indium, there is no metallic indium peak in our samples, demonstrating that the elemental indium exists only in the form of oxide and the major state is In^3+^. The high-resolution XPS spectra of the Ag peak is sketched, where the peak corresponding to metallic silver can be assigned to 374.0 eV (Ag 3d_3/2_) and 368.0 eV (Ag 3d_5/2_) in Fig. 3c, indicating that the Ag species loaded on the surface region are metallic silver.

**Fig. 4:**
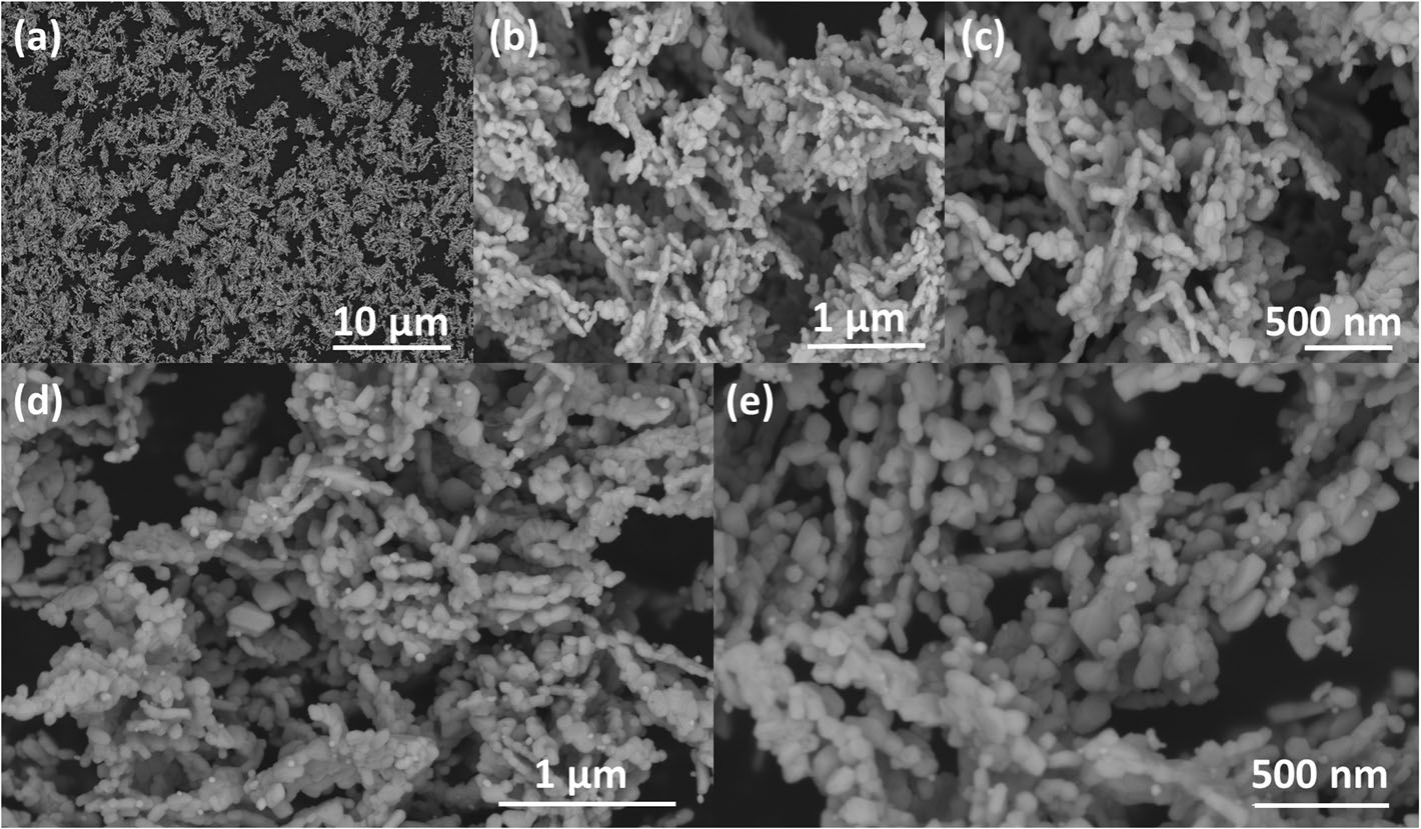
SEM images of pure In_2_O_3_ (**a**, **b**, and **c**) and 5%Ag-In_2_O_3_ (**d** and **e**) samples

The morphology of the pure In_2_O_3_ and 5%Ag-In_2_O_3_ samples was preliminarily demonstrated in Fig. 4a–e by SEM analysis. All samples showed nanograin morphologies with diameters ranged from 20 to 50 nm and ranged from a few hundred nanometers to over 1 μm in lengths. For the pure In_2_O_3_ samples, from Fig. 4a–c, we can see that the surface of each nanograins is smooth. After functionalization processes, we can plainly see that the surface of the In_2_O_3_ nanograins is a little rough in Fig. 4d–e, and that the Ag NPs are distributed on the surface of In_2_O_3_ nanograins. The presented SEM images show that the loading of Ag has no significant effect on the morphology of In_2_O_3_.

**Fig. 5:**
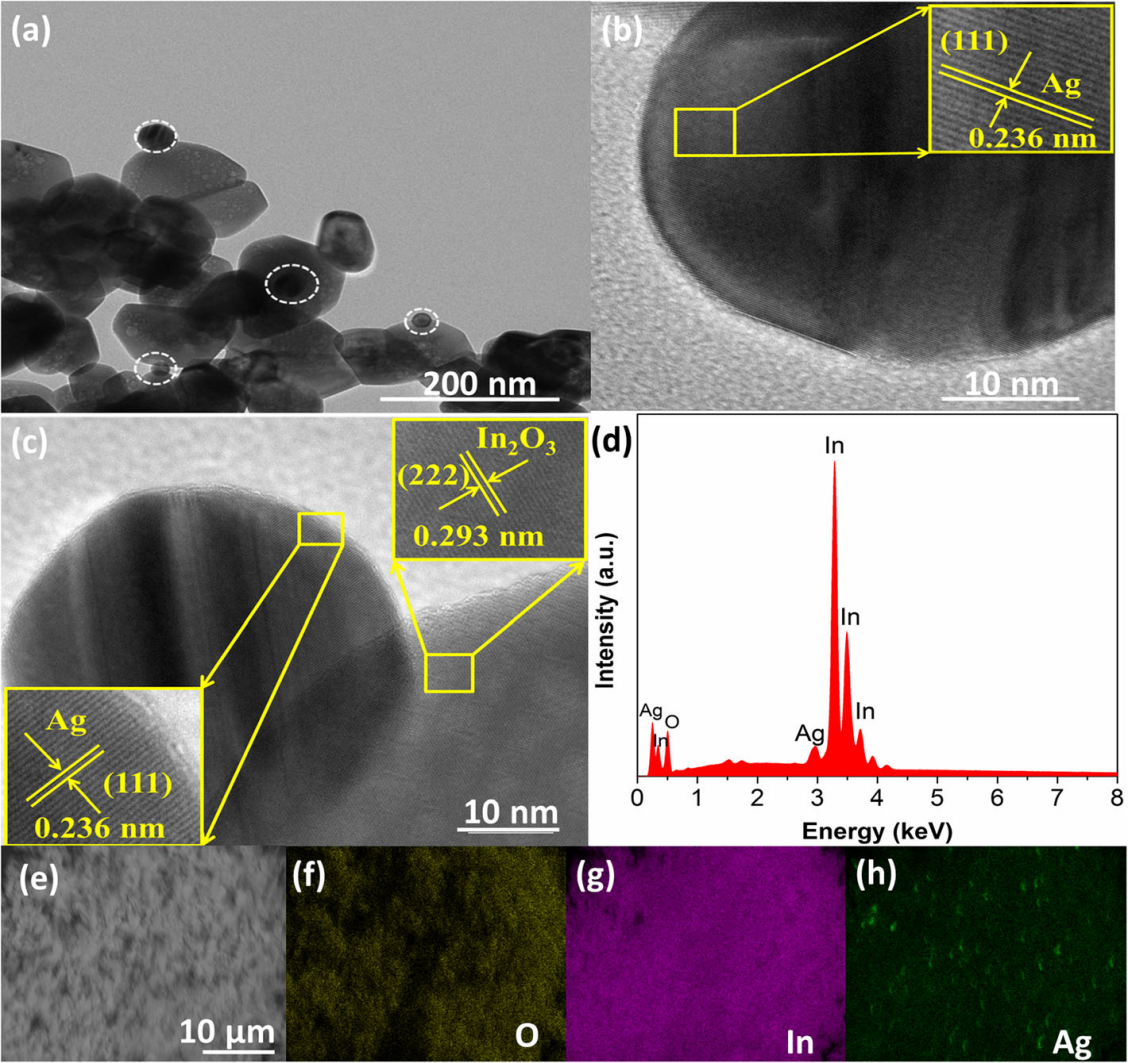
**a** TEM image of 5%Ag-In_2_O_3_ samples. **b**, **c** HRTEM images of 5%Ag-In_2_O_3_ samples. **d** EDS spectra pattern of 5%Ag-In_2_O_3_ samples. **e**–**h** The EDS mapping picture of O, In, and Ag elements of 5%Ag-In_2_O_3_ samples

After Ag nanoparticles are decorated onto the dispersed In_2_O_3_ nanograins, the morphology and crystalline phases of 5%Ag-In_2_O_3_ samples are presented through the TEM images in Fig. 5. It can be seen that Ag NPs with a size from 30 nm to about 100 nm are well pinned on the surfaces of the dispersed In_2_O_3_ nanoparticle, which will be helpful for enhancing gas-sensing properties. In order to further observe the detailed microstructure of In_2_O_3_ and Ag NPs, high-resolution TEM images of the 5%Ag-In_2_O_3_ sample were obtained (Fig. 5b, c). The dispersed In_2_O_3_ are assembled into a single crystal in Fig. 5b and c. The high-resolution TEM images from Fig. 5c show that the lattice plane is 0.293 nm, corresponding to the (222) crystal plane of cubic In_2_O_3_, while the crystal spacing of 0.236 nm is in good agreement with the (111) spacing of Ag. In addition, the interface reveals the existence of strong electronic interaction between In_2_O_3_ nanostructures and Ag nanoparticles.

The energy dispersive X-ray spectroscopy pattern (Fig. 5d) is eloquent proof of the existence of In, O, and a few Ag without any impurity elements. The atomic percentages of In, O, and Ag are 33.99%, 62.43%, and 3.59%, respectively. The atomic ratio of In and O is about 1:2, indicating that 5%Ag-In_2_O_3_ samples are the major phase component in selected region. To further determine the distribution of Ag, the 5%Ag-In_2_O_3_ samples were performed by the EDS. As observed from Fig. 5e–h, the Ag-loaded In_2_O_3_ sample was evenly distributed by elemental mappings of O, In, and Ag, respectively. The results show that there are obvious loads of Ag NPs onto the surface of dispersed In_2_O_3_ nanoparticle and dispersed In_2_O_3_ nanostructure does not accumulate with the decoration of Ag.

**Fig. 6:**
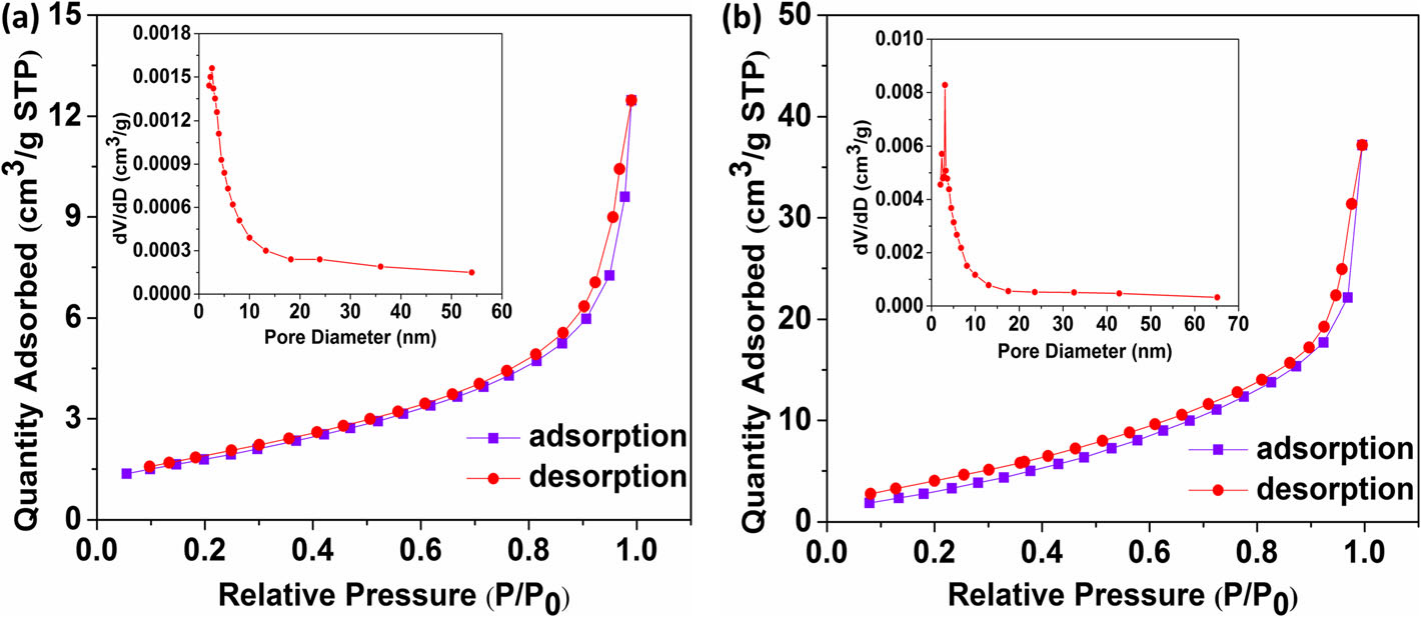
Nitrogen adsorption–desorption isotherms curves of pure In_2_O_3_ (**a**) and 5%Ag-In_2_O_3_ (**b**) samples. Insets are the corresponding pore size distribution curves obtained by the BJH method

In order to obtain the porosity and the specific surface area of the pure In_2_O_3_ and 5%Ag-In_2_O_3_ samples, N_2_ adsorption-desorption experiment method was employed. On the basis of the current IUPAC classification, Fig. 6a and b show the classic type III isotherm to relative pressure (0.1 < P/P_0_ < 1.0) with a type H3 hysteresis loop, which consist of granular material and have no obvious saturated adsorption platform, indicating that the pore structure is very irregular. The pore volume and surface area of 5%Ag-In_2_O_3_ are 0.0650 cm^3^ g^−1^ and 14.4 m^2^ g^−1^ characterized with the Brunauer–Emmett–Teller (BET) method, both of which are larger than the pure In_2_O_3_ (6.5 m^2^g^−1^and 0.0204 cm^3^ g^−1^), demonstrating that the specific surface area gradually raises as being loaded certain content of Ag NPs. The pore-size distribution was measured by using the Barrett–Joyner–Halenda (BJH) way. One can see that the pore sizes of the pure In_2_O_3_ distributes in the range from 2 to 54 nm. For 5%Ag-In_2_O_3_ samples, the pore sizes are all distributed between 2 and 65 nm.

### Gas-Sensing Performance

**Fig. 7:**
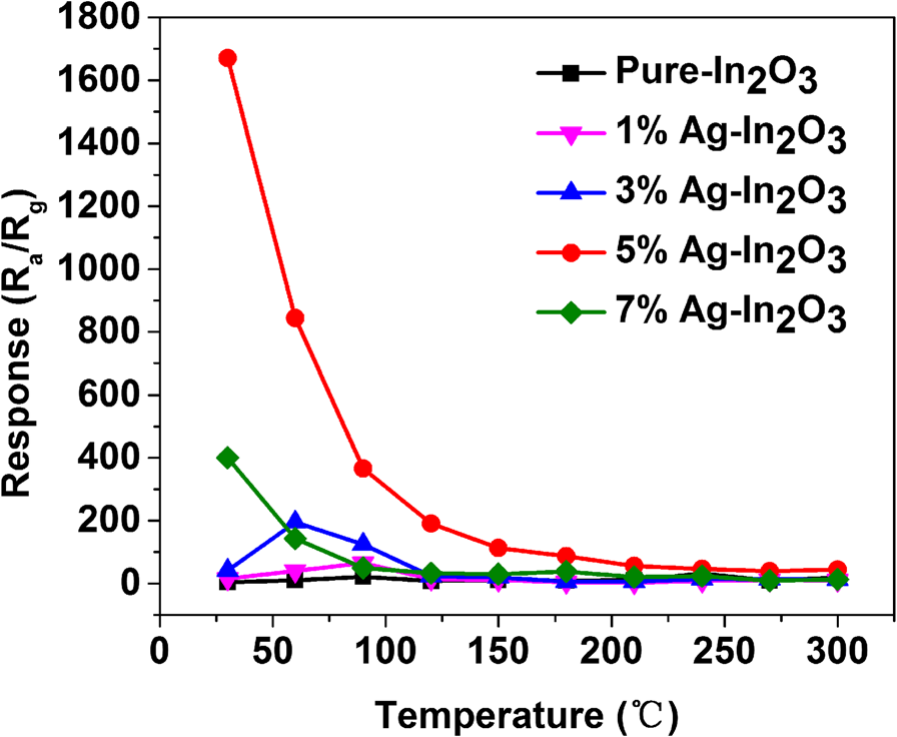
Responses of pure In_2_O_3_, 1%Ag-In_2_O_3_, 3%Ag-In_2_O_3_, 5%Ag-In_2_O_3_, and 7%Ag-In_2_O_3_ gas sensors to 5 ppm gaseous formaldehyde in the operating temperature range from 30 to 300 °C

The gas response upon exposure to 1 ppm HCHO was investigated by increasing the operating temperature of the sensor device to observe the relation between the operating temperature and gas response, and to determine the optimized operating temperature. The pure In_2_O_3_, 1%Ag-In_2_O_3_, 3%Ag-In_2_O_3_, 5%Ag-In_2_O_3_, and 7%Ag-In_2_O_3_ were continuously tested under the 5 ppm gaseous formaldehyde conditions at operating temperatures of 30–300 °C, respectively. The sensing responses of each gas sensor were measured at a fixed temperature, and the recorded values of the gas sensors are shown at room temperature in Fig. 7.

It can be seen that the 5%Ag-In_2_O_3_ sensor has maximum response to formaldehyde gas at 30 °C, and the response is 1670. It tends to increase at lower operating temperatures (5%Ag-In_2_O_3_: 1670, 844, 366, 191, 113, 87, 56, 46.3, 39, and 44.2 at 30, 60, 90, 120, 150, 180, 210, 240, 270, and 300 °C; 7%Ag-In_2_O_3_: 400, 143, 49, 33.1, 29.3, 37.8, 20.3, 23.3, 8.66, and 12.8 at the same operating temperatures). The gas responses of the Ag-In_2_O_3_ sensors show higher values than pure In_2_O_3_ in all operating temperature ranges, which can fix the optimal operating temperature and the optimum HCHO response of the sensors. Among them, it can be clearly seen that 5%Ag-In_2_O_3_ sensor has the highest responses (1670) to 5 ppm HCHO at room temperature demonstrating the superior sensing properties of the sensor, which is higher than other sensors. The reason the higher gas response appears at room operating temperatures can be attributed to Ag NPs catalytic (spill-over effect) and electronic (generation of schottky barrier) sensitization. After loading the Ag NPs, the operating temperature is reduced and the formaldehyde sensitive response enhanced significantly. Nevertheless, with the loading of Ag NPs further enhancing, the response value decreases. This can be ascribed to the reduction in number of surface active sites of In_2_O_3_, which indicates that the excessive coverage of Ag NPs and the permeability of gas are affected, and then the catalytic action of Ag NPs is weaken, causing a decrease in adsorbed oxygen ions [28]. Compared with other previously reported gas sensors based on In_2_O_3_ sunflower, In_2_O_3_/ZnO nanocomposites, or In_2_O_3_ nanorods, our gas sensor shows notable gas response at room temperature [28, 29, 40].

**Fig. 8:**
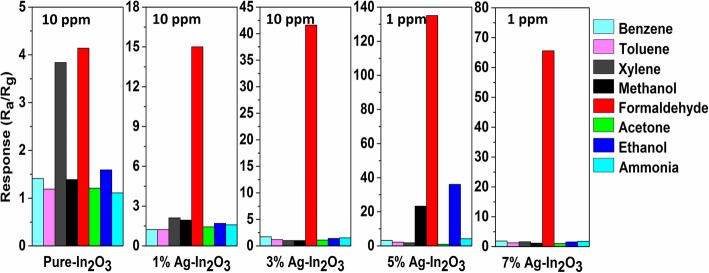
The gas response of pure In_2_O_3_, 1%Ag-In_2_O_3_, and 3%Ag-In_2_O_3_ (benzene, toluene, xylene, methane, formaldehyde, acetone, ethanol, and ammonia) with a concentration of 10 ppm at 30 °C, 5%Ag-In_2_O_3_ and 7%Ag-In_2_O_3_ toward this gases with a concentration of 1 ppm at 30 °C

To further confirm the selectivity of the synthesized gas sensors toward HCHO, the selectivity sensing performance of pure In_2_O_3_, 1%Ag-In_2_O_3_, 3%Ag-In_2_O_3_, 5%Ag-In_2_O_3_, and 7%Ag-In_2_O_3_ sensors was tested at room temperature toward 10 ppm of several volatile organic compounds, including benzene, toluene, xylene, methane, formaldehyde, acetone, ethanol, and ammonia, 5%Ag-In_2_O_3_ and 7%Ag-In_2_O_3_ toward this gases with a concentration of 1 ppm at room temperature. As shown in Fig. 8, the Ag-In_2_O_3_ sensors demonstrate superior selectivity to formaldehyde, whereas they have poor responses to other typical interference gases at the same temperature, especially the 5%Ag-In_2_O_3_ sensor. This indicates that the prepared sensor has quite excellent selectivity towards formaldehyde.

**Fig. 9:**
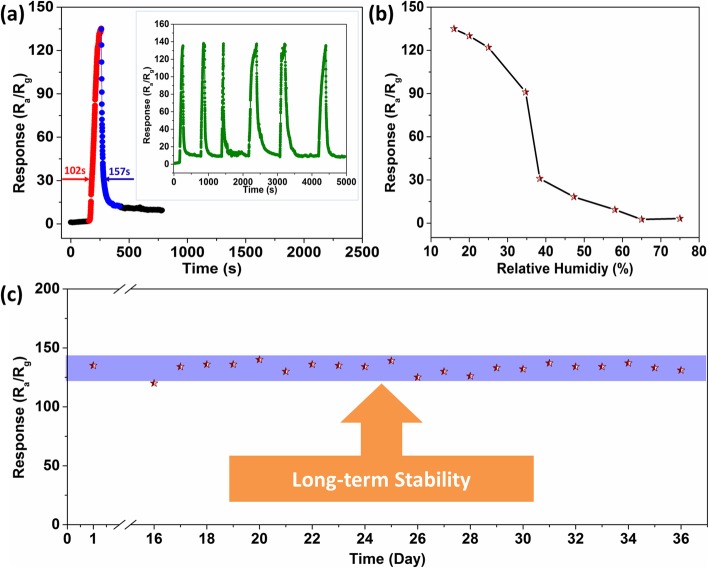
**a** The response–recovery of the 5%Ag-In_2_O_3_ toward 1 ppm HCHO gas for 6 cycles at 30 °C. **b** Responses of 5%Ag-In_2_O_3_ sensor toward 1 ppm of HCHO under different humidity conditions at 30 °C. **c** Long-term stability tests of 5%Ag-In_2_O_3_ sensor toward 1 ppm HCHO after continuous evaluation for 36 days at 30 °C

Furthermore, the stability of the 5%Ag-In_2_O_3_ sensor is shown in Fig. 9. The 5%Ag-In_2_O_3_ sensor was investigated toward 1 ppm HCHO for 6 cycles at room temperature (Fig. 9a), which demonstrates the excellent reproducibility to HCHO at room temperature. As demonstrated in Fig. 9c, the 36-day response test results show that the 5%Ag-In_2_O_3_ sensor not only possesses high gas-sensing performance but also excellent long-term stability. Meanwhile, the gas-sensing properties of 5%Ag-In_2_O_3_ sensor under different humidity conditions were investigated (Fig. 9b). Obviously, the sensor has not been significant affected on the sensing performance under a relative humidity range of 10−30%. Nevertheless, when the relative humidity range increases from 30 to 80%, the gas-sensing properties begin to reduce gradually.

**Fig. 10:**
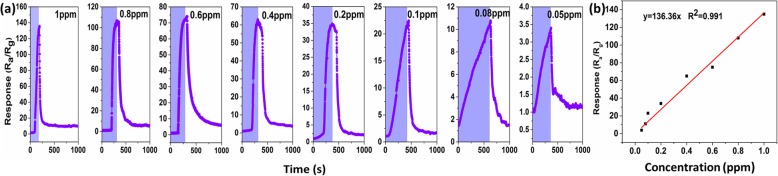
**a**, **b** Real-time response–recovery characteristic curve of the 5%Ag-In_2_O_3_ toward formaldehyde at different concentrations (1, 0.8, 0.6, 0.4, 0.2, 0.1, 0.08, 0.05 ppm) at 30 °C

The real-time dynamic gas responses of the 5%Ag-In_2_O_3_ sensors toward HCHO at various concentrations at room temperature are presented in Fig. 10. The responses to 1, 0.8, 0.6, 0.4, 0.2, 0.1, 0.08, and 0.05 ppm formaldehyde were calculated to be R_a_/R_g_ = 135, 108, 75, 65, 34, 23, 11, and 3.85, respectively. The sensitivity amplitude increases monotonically with gas concentration and is far from saturation until the gas concentration reaches 0.05 ppm, which is beneficial to the quantitative measurement of formaldehyde. Notably, the response is still as high as 3.85 when sensor is exposed to concentrations of formaldehyde as low as 0.05 ppm, indicating the ultra-low detection concentration of the sensor.

### Mechanism of the Gas Sensor

**Fig. 11:**
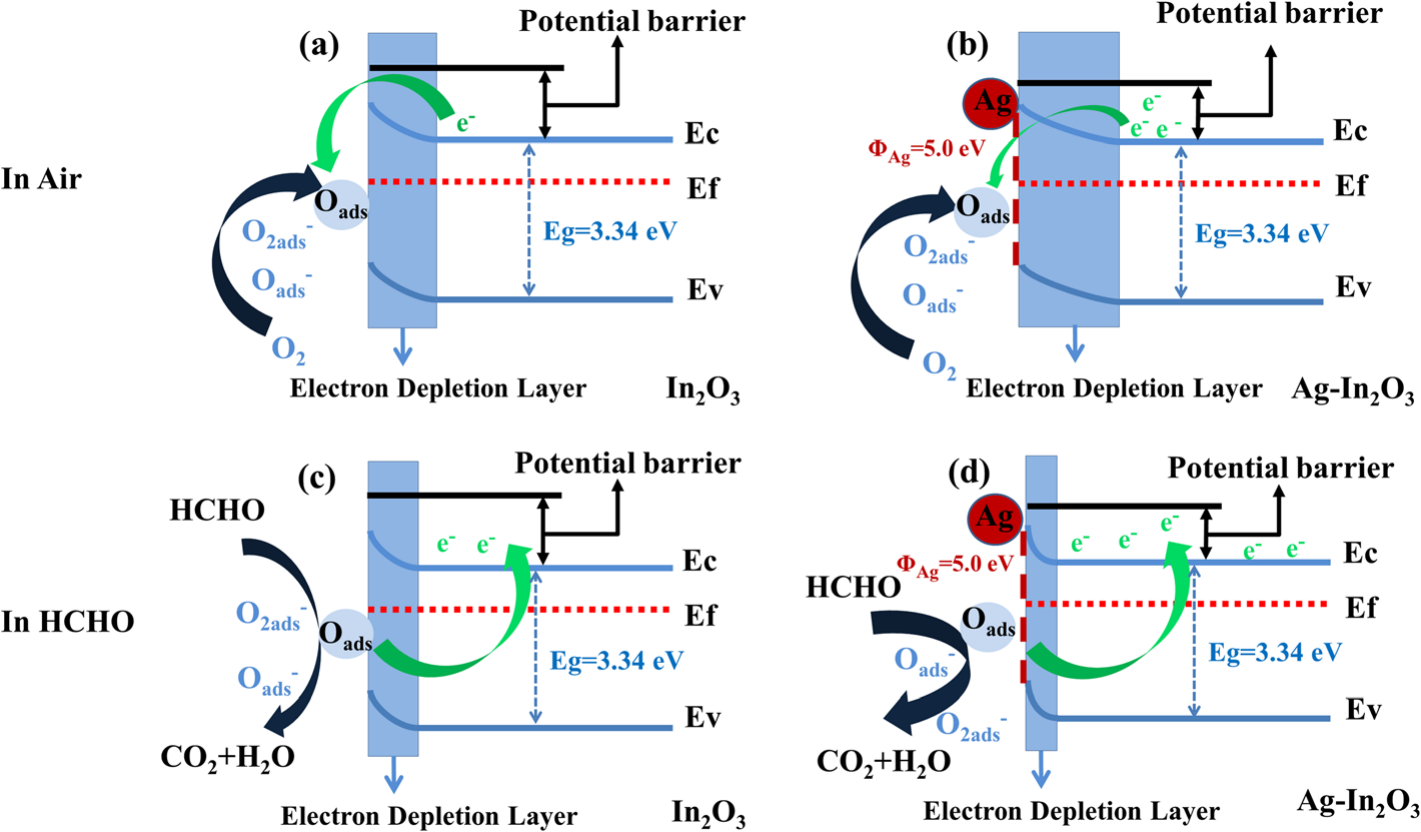
**a**–**d** HCHO sensing mechanism of schematic illustration for 5%Ag-In_2_O_3_ and pure In_2_O_3_, respectively

The In_2_O_3_ semiconductor is a chemical resistance–sensing material, and its electrical property changes mainly with the reaction of HCHO on the surface of In_2_O_3_. A HCHO sensing schematic diagram is shown in Fig. 11. When the sensor is exposed to air, an abundance of oxygen molecules in air will be absorbed onto the surface of the In_2_O_3_, and this oxygen captures electron from the material’s conductive band and converts them into more active chemical adsorbed oxygen, thereby creating a space charge area (depletion layer) that greatly increases initial resistance. The electron depletion layer has a great influence on the initial resistance of the sensor in the air. The major forms of chemisorbed oxygen species are O_2_^−^ and O^−^, which can be described as Eqs. (1)-(3):

1 $$ {O}_{2(gas)}\to {O}_{2(ads)} $$

2 $$ {O}_{2(ads)}+{e}^{-}\to {{O_2}^{-}}_{(ads)} $$

3 $$ {{O_2}^{-}}_{(ads)}+{e}^{-}\to 2{O^{-}}_{(ads)} $$

When the sensor is placed in an environment inflated with HCHO, the chemical adsorption oxygen reacts with HCHO, discharging electrons back to the conductive band, reducing the space charge area thickness, and thus decreasing the sensor resistance. The occurred reaction can be explained as followed in Eqs. (4) and (5):

4 $$ HCHO+{{O_2}^{-}}_{ads}\to C{O}_2+{H}_2O+{e}^{-} $$

5 $$ HCHO+2{O^{-}}_{(ads)}\to C{O}_2+{H}_2O+2{e}^{-} $$

**Fig. 12:**
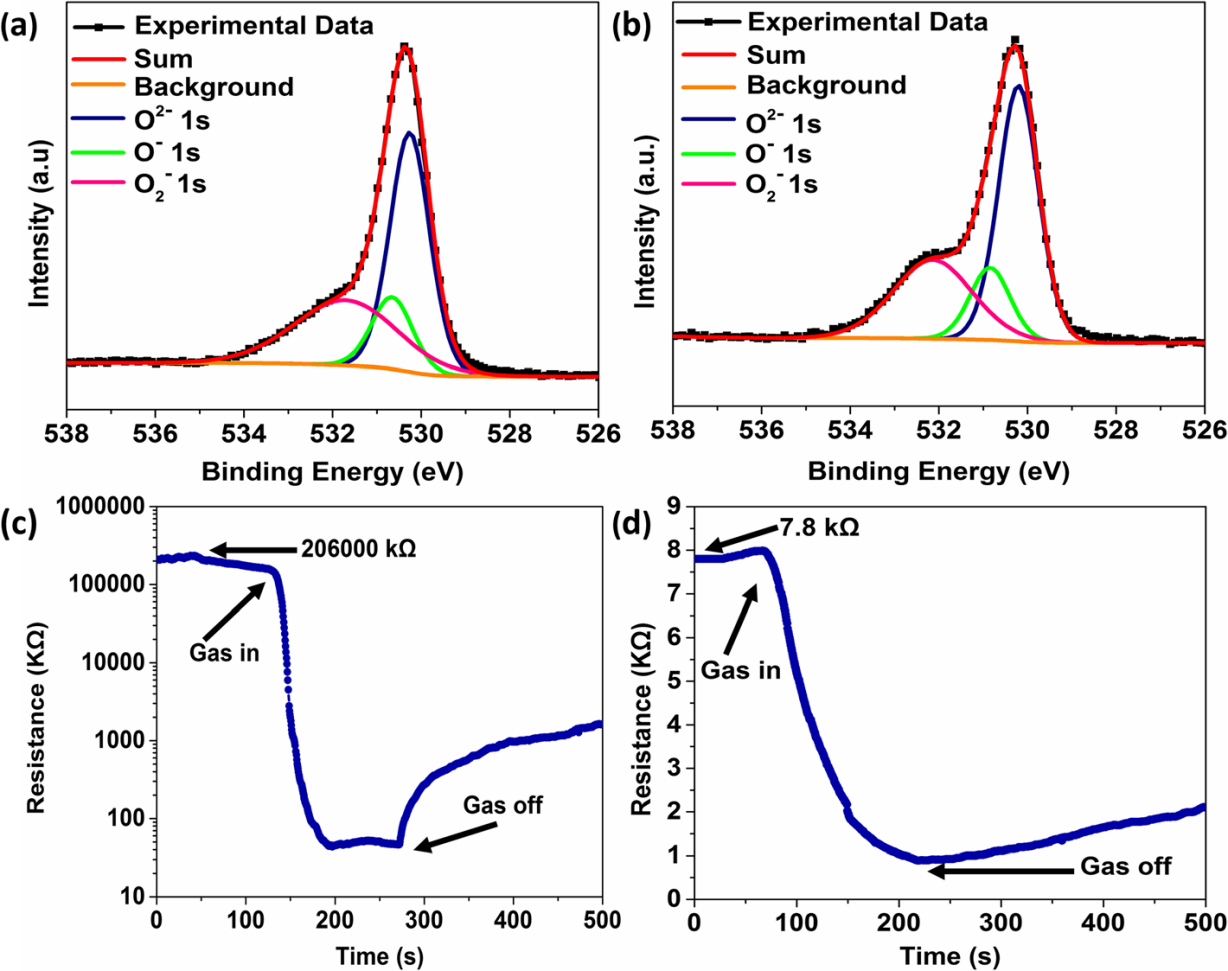
XPS spectra of 5%Ag-In_2_O_3_ (**a**) and pure In_2_O_3_ (**b**) in the vicinity of O1s. Dynamic resistance transition characteristics of the 5%Ag-In_2_O_3_ (**c**) and pure In_2_O_3_ (**d**) toward 40 ppm of formaldehyde at 30 °C

Obviously, the sensor performances based on 5%Ag-In_2_O_3_ are much higher than those of pure In_2_O_3_. This excellent response is ascribed to the electronic sensitization and chemical effect of Ag NPs. The Ag NPs have high availability for the catalytic activation of the dissociation of molecular oxygen, and the activated oxygen species created are then spilled onto the metal oxides surface and interact with the adsorption-desorption reactions of oxygen [42]. The chemisorbed oxygen plays a critical role in the gas sensing of sensors by regulating the reaction with tested gases [43]. Pure In_2_O_3_ and 5%Ag-In_2_O_3_ based on sensors were investigated by XPS to confirm the ratio of the chemisorbed oxygen in the samples. The O_1_ spectra of the pure In_2_O_3_ and 5%Ag- In_2_O_3_ (Fig. 12a, b and Table 1) show that the adsorbed oxygen content (2.46% of O^−^ and 19.54% of O_2_^−^) of 5%Ag- In_2_O_3_ is higher than that of pure In_2_O_3_ (1.83% of O^−^ and 16.05% of O_2_^−^), which is mainly due to the Ag Nps spill-over effect on metal oxide semiconductors [44]. Owing to the high conductivity and catalytic properties of Ag NPs [28, 42, 45, 46], Ag NPs on the surface of the metal oxides enhance the chemical activity of the chemisorbed oxygen species and spill the oxygen species over the substrate, which accelerates for gas sensing at low temperature.

**Table 1:** The ratio of chemical component of 5%Ag-In_2_O_3_ and pure In_2_O_3_.

Content	O^2−^	O^−^	O_2_^−^
5%Ag-In_2_O_3_	78.00	2.46	19.54
pure In_2_O_3_	82.13	1.83	16.05

Moreover, the Schottky junction can be formed at the interface between In_2_O_3_ and Ag due to the difference in band gap and work function [47, 48]. When the 5%Ag-In_2_O_3_ material is exposed to the atmosphere, compared with pure In_2_O_3_, the depletion region in 5%Ag-In_2_O_3_ composites is further broadened due to the presence of Schottky junction between Ag and In_2_O_3_ interface. The charged species such as O^−^ and O^2−^ adsorbed on the surface of In_2_O_3_ also contribute to electron depletion by capturing free electrons from the sensing materials [15] (Fig. 12a, b). The base resistance of 5%Ag-In_2_O_3_ was investigated to 206000 kΩ, far higher than the resistance (7.8 kΩ) of pure In_2_O_3_ (Fig. 12c, d), further demonstrating that the Ag NPs can remarkably enhance baseline resistance. When the 5%Ag-In_2_O_3_ material is exposed to HCHO in the sensing process, the Schottky junction forming at the interface between Ag and In_2_O_3_ produces more overflow electrons and donates it to the In_2_O_3_ matrix, resulting in efficient modulation of the depletion layer. Besides, with more oxygen substances adsorbed on the surface of Ag/In_2_O_3_, the redox reactions occurred between HCHO and chemical adsorbed oxygen are enhanced. The redundant electrons generated by these increased surface reactions result in a greater reduction in resistance of the 5%Ag-In_2_O_3_-based sensors in HCHO (Fig. 12c, d). Hence, 5%Ag-In_2_O_3_ sensor possesses superior sensing performance to HCHO.

## Conclusion

In summary, we realized an ultra-high performance HCHO chemiresistor with Ag nanoparticles sensitized dispersed In_2_O_3_ semiconductor. The 5%Ag-In_2_O_3_ sensor demonstrates ultra-high response (135), short response time (102 s) and recovery time (157 s) to 1 ppm HCHO gas, and an ultra-low detection concentration (0.05 ppm) at room temperature. Compared with other HCHO sensors, the sensor has good reproducibility and strong responsivity at room temperature, and will have an excellent practical application prospect.

## Data Availability

The datasets supporting the conclusions of this article are included within the article, and further information about the data and materials could be made available to the interested party under a motivated request addressed to the corresponding author.

## Abbreviations

Ag NPs: Ag nanoparticles
EDS: Energy dispersive X-ray spectroscopy
HCHO: Formaldehyde
HRTEM: High-resolution transmission electron microscopy
ppm: Parts-per-million
Ra: Resistance in air
Rg: Resistance in target gas
SEM: Scanning electron microscopy
TEM: Transition electron microscopy
VOCs: Volatile organic compounds
XPS: X-ray photoelectron spectroscopy
XRD: X-ray powder diffraction
